# Exploring COX-Independent Pathways: A Novel Approach for Meloxicam and Other NSAIDs in Cancer and Cardiovascular Disease Treatment

**DOI:** 10.3390/ph17111488

**Published:** 2024-11-06

**Authors:** Lixia Cheng, Zhenghui Hu, Jiawei Gu, Qian Li, Jiahao Liu, Meiling Liu, Jie Li, Xiaowen Bi

**Affiliations:** 1 Department of Medical Genetics and Cell Biology, School of Basic Medical Sciences, Jiangxi Medical College, Nanchang University, Nanchang 330006, China; chenglixia99@163.com (L.C.); huzhenghui88@163.com (Z.H.); 15736745922@163.com (Q.L.); liujiahao4035@163.com (J.L.); 13517180571@163.com (M.L.); L364210lijie@163.com (J.L.); 2 Department of Precision Genomics, Intermountain Healthcare, 5121 Cottonwood St., Murray, UT 84107, USA; jiawku17@gmail.com

**Keywords:** Meloxicam, NSAIDs, cancer, cardiovascular disease, COX-independent pathway

## Abstract

As a fundamental process of innate immunity, inflammation is associated with the pathologic process of various diseases and constitutes a prevalent risk factor for both cancer and cardiovascular disease (CVD). Studies have indicated that several non-steroidal anti-inflammatory drugs (NSAIDs), including Meloxicam, may prevent tumorigenesis, reduce the risk of carcinogenesis, improve the efficacy of anticancer therapies, and reduce the risk of CVD, in addition to controlling the body’s inflammatory imbalances. Traditionally, most NSAIDs work by inhibiting cyclooxygenase (COX) activity, thereby blocking the synthesis of prostaglandins (PGs), which play a role in inflammation, cancer, and various cardiovascular conditions. However, long-term COX inhibition and reduced PGs synthesis can result in serious side effects. Recent studies have increasingly shown that some selective COX-2 inhibitors and NSAIDs, such as Meloxicam, may exert effects beyond COX inhibition. This emerging understanding prompts a re-evaluation of the mechanisms by which NSAIDs operate, suggesting that their benefits in cancer and CVD treatment may not solely depend on COX targeting. In this review, we will explore the potential COX-independent mechanisms of Meloxicam and other NSAIDs in addressing oncology and cardiovascular health.

## 1. Introduction

Inflammation, as a complex biological process, is often considered the body’s response to microbial infection, tissue damage, or other cellular stressors. It acts as an acute protective mechanism aimed at maintaining internal stability. However, prolonged immune responses associated with chronic inflammation have been implicated in the development of various malignant tumors [1]. Alterations in the tumor microenvironment brought about by chronic inflammation are associated with various steps involved in tumor development, including cell survival, proliferative transformation, invasion, angiogenesis, and metastasis, among others [2]. Chronic inflammation recruits a large number of inflammatory cells, cytokines, reactive oxygen species (ROS), and reactive nitrogen species (RONS), which can promote cancer cell invasion and metastasis by stimulating their survival, proliferation, and angiogenesis [2]. In the context of tumorigenesis mechanisms, the accumulation of ROS and RONS leads to DNA damage and interferes with DNA repair, further promoting the development of cancer; simultaneously, DNA damage triggers a positive feedback loop that enhances inflammation [3]. This cycle creates a positive feedback loop: the prolonged inflammatory response alters the tumor microenvironment, while metabolic changes, oxidative stress, and cell death induced by the tumor itself perpetuate inflammation.

In addition, several lines of evidence suggest that chronic inflammation is involved in the development of cardiovascular disease (CVD). Studies have shown that the elevation of inflammatory cytokines (IL-1, IL-6, and TNF-α) contributes to a state of chronic inflammation in the cardiovascular system, promoting conditions like myocardial infarction, hypertension, atherosclerosis, and hypertrophic heart failure [4]. Therefore, inflammation has been identified as one of the independent risk factors for CVD. Furthermore, diseases such as diabetes, obesity, and rheumatoid arthritis are also significant contributors to CVD due to their associated inflammatory signals. Interestingly, physiological conditions leading to vascular cellular senescence can induce a sterile low-grade inflammatory state, which accelerates vascular aging and increases the risk of diseases such as atherosclerosis, myocardial infarction, and heart failure [5,6].

Therefore, inflammation can be regarded as a common risk factor for both cancer and CVD, making it a potential target for the prevention and treatment of these conditions [7]. Non-steroidal anti-inflammatory drugs (NSAIDs) are a chemically broad class of drugs that are widely used in clinical practice for their anti-inflammatory, analgesic, and antipyretic properties. The discovery of the first NSAIDs, salicylates, dates back to 1829, followed by the introduction of Aspirin and Indomethacin, which led to the rise of many other NSAIDs [8]. Their anti-inflammatory activity is primarily mediated through the inhibition of cyclooxygenase 1 (COX-1) and cyclooxygenase 2 (COX-2), an enzyme that converts arachidonic acid (ARA) into prostaglandins (PGs), thereby promoting the development of inflammation [9], cancer [10], and a variety of CVDs [11]. Consequently, NSAIDs are frequently employed in the prevention and treatment of a range of conditions, including rheumatoid arthritis, ankylosing spondylitis, cancer, and CVD. While NSAIDs generally exhibit comparable efficacy in clinical settings, subtle differences among them (Table 1) and the varying patient responses to NSAIDs (side effects) have influenced patients’ choice of medication. Meloxicam, a selective COX-2 inhibitor, was initially developed in Germany to reduce the gastrointestinal damage associated with other conventional NSAIDs [12]. First marketed in Germany in 1996, it is now approved for the treatment of rheumatoid arthritis and osteoarthritis in several countries, including the United States and France [13]. As a new type of NSAID, Meloxicam has also demonstrated strong inhibitory effects on COX; unlike traditional NSAIDs, its inhibitory effect on the induced COX-2 is much greater than that of the constitutive COX-1, which is often linked to gastric and renal impairments and other adverse effects [12]. Clinical trials have shown that Meloxicam is comparable to piroxicam and naproxen in treating osteoarthritis, but it has a notably lower incidence of gastrointestinal and renal complications [13,14]. In addition, Meloxicam has been clinically utilized in the treatment of tumors. In 1998, researchers discovered that meloxicam could inhibit COX-2, which is highly expressed in colorectal cancer [15]. Recent studies have shown that COX-2 is also significantly expressed in other cancers, including liver cancer [16,17], esophageal squamous carcinoma [18], and osteosarcoma [19]. Meloxicam inhibits the growth, invasiveness, and metastasis of cancer cells by downregulating COX-2 expression [16,17,18,19].

As a class of anti-inflammatory drugs, NSAIDs are often used in the prevention or treatment of cancer and CVD with positive outcomes. Research indicates that Meloxicam and some other NSAIDs (such as Diclofenac, Ibuprofen, Sulindac, and ABT-346) can promote the apoptosis of cancer cells through COX-dependent and COX-independent pathways [16,20,21,22], thereby inhibiting the growth and proliferation of cancer cells and exerting their anti-tumor activity. At the same time, Diclofenac and Aspirin can inhibit cancer metastasis and recurrence through COX-PGE pathways [23] or other non-COX mechanisms (for example, Diclofenac can target CD37) [24]. Clinically, targeting inflammation with anti-tumor drugs is a common strategy, and several NSAIDs, including Aspirin, Ibuprofen, Indomethacin, Sulindac, and Meloxicam, have demonstrated the ability to reduce cancer risk factors, morbidity, and mortality in various cancers such as colorectal, breast, and lung cancer [25,26]. In the context of CVD, NSAIDs also play a significant role, with Aspirin noted for its superior cardioprotective effects [27]. In addition, in our and other studies, we found that Meloxicam not only targets COX-2, but also targets other signaling pathways, including MAPKs [28], NF-κB [29], Wnt/β-catenin, and PI3K/ATK [30] signaling pathways, all of which are associated with the development of cancer and CVD. Meanwhile, other NSAIDs have also been reported to target these signaling pathways. Therefore, this article reviews the COX-independent pathways of selective COX-2 inhibitor Meloxicam and other NSAIDs in the prevention and treatment of cancer and CVD, offering new insights into their therapeutic potential.

**Table 1:** Classification, structure, and targets of action of NSAIDs. Drug structure diagrams quoted in “Drug Library” (https://go.drugbank.com, accessed on 7 September 2024).

	Selectivity for COXs	Drug	Structure	Target	Reference
NSAIDs	COX-1 and COX-2	Aspirin	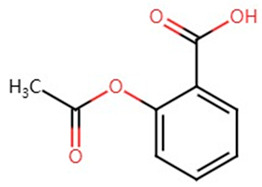	Breast Cancer, Colon and Rectal Cancer, Lung Cancer, Prostate Cancer, Stomach Cancer, Bile Duct Cancer, Melanoma	[23,25,26,31,32,33,34,35,36,37,38,39,40]
		Ibuprofen	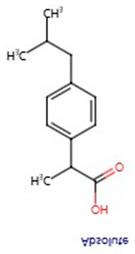	Breast Cancer, Bile Duct Cancer, Anaplastic Thyroid Cancer	[41,42,43]
		Sulindac	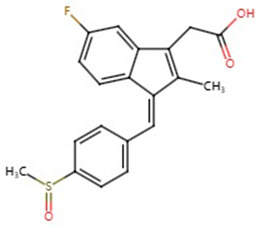	Colon and Rectal Cancer, Lung Cancer, Breast Cancer	[21,42,44,45,46]
		Sulfasalazine	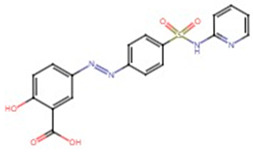	Lung Adenocarcinoma, Esophageal Cancer, Breast Cancer	[47,48,49,50]
		Indomethacin	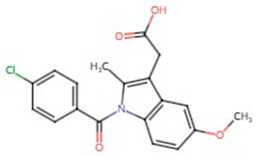	Large B-cell Lymphoma, Breast Cancer	[42,51]
		Ketoprofen	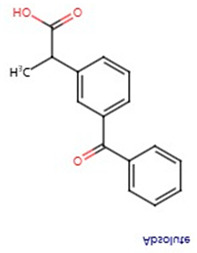	Breast Cancer	[52]
	COX-2 selective	Meloxicam	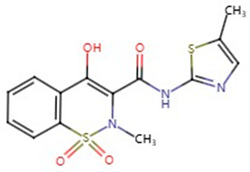	Liver Cancer, Multiple myeloma, Colorectal cancer, Esophageal cancer, Osteosarcoma	[15,16,18,19,53,54]
		Lornoxicam	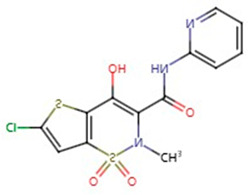	Melanoma	[23]
		Celecoxib	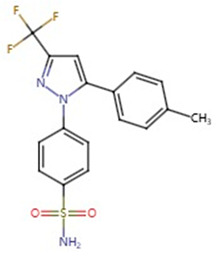	Lung Cancer, Pancreatic Cancer, Liver Cancer, Glioblastoma, Rhabdomyosarcoma	[32,44,55,56,57,58,59,60]
		Firocoxib	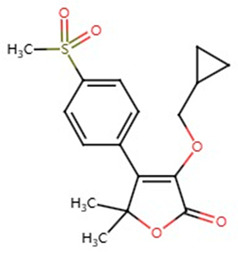	Breast Cancer	[61]
		Diclofenac	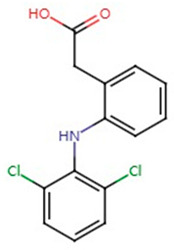	Large B-cell Lymphoma, Breast Cancer, Pancreatic Cancer, Cholangiocarcinoma, Liver cancer, Vulvar squamous cell carcinoma	[22,24,41,42,56,62,63]

## 2. COX-Dependent Pathways

**Figure 1:**
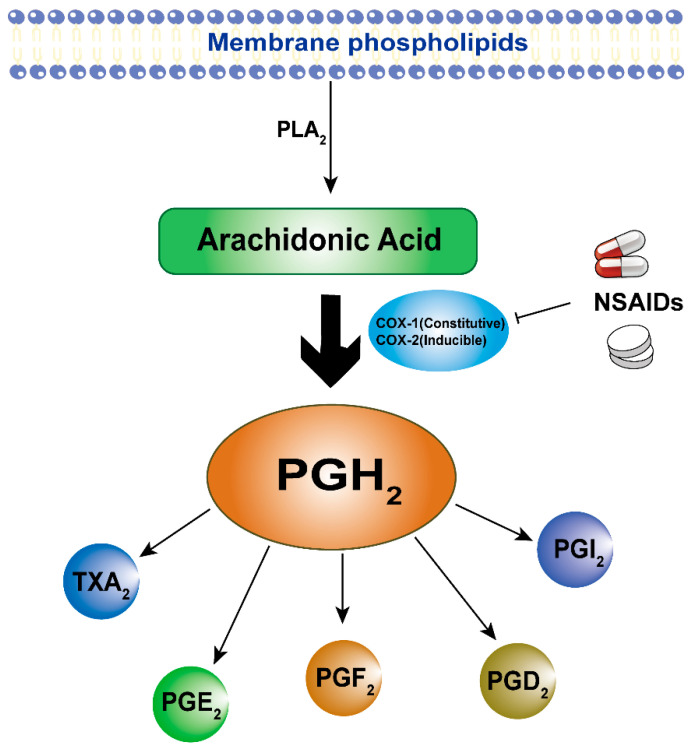
Overview of NSAIDs targeting COX in response to tumor and CVD pathways. As a substrate for the action of cyclooxygenase, ARA is catalyzed by phospholipases from membrane phospholipids. COX is a key enzyme in the metabolism of ARA, with two isoforms, structural (COX-1) and inducible (COX-2), and NSAIDs block prostaglandin synthesis, which is involved in cancer and CVD, through inhibition of the enzymatic activity of COX. Abbreviations: Phospholipases A_2_, PLA_2_; Prostaglandin H2 synthase, PGH2; Thromboxane A_2,_ TXA_2_; Prostaglandins (respective receptors): prostaglandins E_2_ (PGE_2_), prostaglandins F_2_ (PGF_2_), prostaglandins D_2_ (PGD_2_), and prostaglandins I_2_ (PGI_2_).

There are two isoforms of COX: COX-1 and COX-2. Among them, COX-2 is a membrane-bound and rate-limiting enzyme that plays a role in the development of diseases such as inflammation [9], cancer [64], and CVDs [65,66] by participating in the synthesis of PGs, which are induced to be produced in large quantities under pathological conditions. It is also for this reason that COX has long been known as a target for the treatment of inflammation and the prevention and treatment of tumors and CVD. Most NSAIDs exert their effects by inhibiting the enzymatic activity of COX-2, thereby blocking PG synthesis. However, conventional NSAIDs (such as Diclofenac, Ibuprofen, and Naproxen) can inhibit COX-1 activity in addition to COX-2 activity [41,67,68] (Figure 1). As a key enzyme in the synthesis of thromboxane A2 (TXA_2_), COX-1 is stably expressed in most tissues and maintains the normal physiological functions of tissues. Especially in the gastric and intestinal mucosal epithelium, COX-1 and its product PG can play a strong protective role [69]. In contrast, COX-1 can promote platelet aggregation and vasoconstriction through TXA_2_, thereby increasing the risk of CVD [70]. Aspirin, in addition to inhibiting inflammation-induced COX-2, irreversibly inhibits COX-1, thereby reducing inflammation and the risk of thrombosis to prevent CVD [71]. Unfortunately, the inappropriate use of Aspirin and other non-selective NSAIDs can lead to side effects such as gastric injury, gastric bleeding, and gastric ulcers due to COX-1 inhibition [72]. This has led to the development of selective COX-2 inhibitors like Meloxicam, which aim to alleviate COX-1-related side effects, although they can still cause harm. In recent years, studies have found that both traditional NSAIDs and new selective COX-2 inhibitors have COX-2 independent pathways of action. Therefore, it is of great significance to explore the COX-independent pathways of NSAIDs.

## 3. COX-2-Independent Anti-Cancer Pathways

### 3.1. Protein Targets of Meloxicam and Other NSAIDs Against Cancer

#### 3.1.1. AXL Receptor Tyrosine Kinase (AXL)

**Figure 2:**
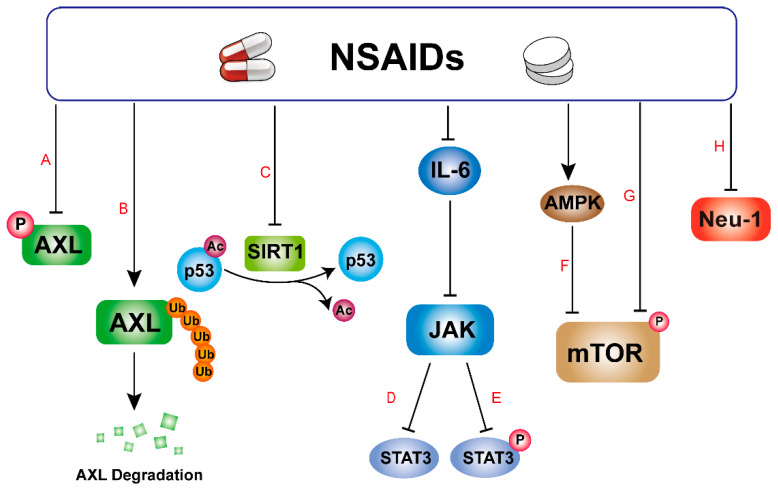
Protein targets of Meloxicam and other NSAIDs against cancer. (A) NSAIDs inhibit the phosphorylation of AXL. (B) NSAIDs promote ubiquitinated degradation of AXL. (C) NSAIDs inhibit the deacetylase activity of SIRT1. (D,E) The protein expression and phosphorylation of STAT3 were inhibited by NSAIDs. (F,G) NSAIDs inhibit the phosphorylation of mTOR, in part by activating the AMPK pathway. (H) NSAIDs inhibit the enzymatic activity of Neu-1.

As a receptor kinase, AXL was initially identified as a transforming gene in chronic granulocytic leukemia, but with deeper research, it has been gradually discovered that AXL is also able to participate in the regulation of a variety of responses in tumor cells, including cell proliferation, survival, and migration [73]. AXL exhibits high or ectopic expression in multiple malignant tumors [74], and this overexpression has been linked to drug resistance in lung, breast, and esophageal cancers [75], as well as tumor invasion [76]. With the in-depth study of AXL, it has been found that AXL can become a rising star of cancer therapeutic targets [73], and some NSAIDs are able to target AXL to achieve anti-tumor effects (Figure 2A,B). For instance, Lornoxicam (a selective COX-2 inhibitor), Aspirin, and Acetaminophen can inhibit melanoma metastasis and recurrence by inhibiting the formation of the Hsp90-CDC37 complex, leading to the misfolding of AXL, thus initiating the ubiquitination–proteasome system (UPS) to degrade AXL [23]. Additionally, Sulfasalazine (SAS), a synthetic NSAID, can inhibit drug resistance and invasiveness in lung adenocarcinoma by blocking AXL phosphorylation [47].

#### 3.1.2. NAD-Dependent Deacetylase 1 (SIRT1)

SIRT1 is a NAD^+^-dependent deacetylase that has been implicated in the occurrence and progression of various cancers, including gastric cancer [77], breast cancer [78], colon cancer [79], prostate cancers, and some certain hematopoietic malignancies [80]. Research indicates that SIRT1 influences key processes such as cell proliferation, migration, invasion, and colony formation, thereby mediating tumor development [77,78,79,80] and contributing to drug resistance [81]. Notably, the use of SIRT1 inhibitors has been shown to significantly reverse its promoting effects on tumor progression [82].

In addition to specific inhibitors, many NSAIDs such as Meloxicam, Aspirin, Celecoxib, and Diclofenac can also target SIRT1 (Figure 2C). Aspirin increases SIRT1 expression, promoting cellular senescence in colon cancer cells and exerting anti-tumor activity [31]. Conversely, Celecoxib and Sulindac inhibit SIRT1 expression, thereby reducing the migration and invasion of lung cancer cells [44]. Meloxicam, when combined with Filgrastim, can enhance the success rate of autologous stem cell transplantation (ASCT) in patients with multiple myeloma (MM) by reducing the oxidative stress of hematopoietic progenitor cells, an inhibitory effect that is most likely mediated by SIRT1, thereby increasing the number of hematopoietic stem cells (PBSC) collected [54,62]. In addition, Diclofenac exerts its anti-tumor effects by targeting SIRT1 to promote p53 acetylation and increase p21 expression [62].

#### 3.1.3. Signal Transducer and Activator of Transcription 3 (STAT3)

STAT3 is a multifaceted transcriptional regulator that plays a crucial role in various biological processes, including cell growth, differentiation, and maturation [83]. It is recognized as an oncogene in tumor development, with abnormal STAT3 expression reported in nearly 70% of cancers [84]. Over-activated STAT3 promotes the activation of oncogenic cytokines and growth factor receptors and induces the overproduction of cytokines such as IL-6 and EGF, which in turn promotes tumor invasion, migration, metastasis, and angiogenesis [85]. Given its significant role in cancer progression, researchers are focusing on developing STAT3 inhibitors for cancer treatment. In addition to addressing intrinsic cellular changes, the evolving immune environment poses a considerable challenge in cancer therapy. STAT3 inhibitors have shown promise in suppressing the growth of various cancers [86] while also targeting inflammation [87]. Interestingly, some NSAIDs have been found to affect STAT3 as well (Figure 2D,E). For instance, Celecoxib inhibits cancer cell proliferation and migration by down-regulating both the expression and phosphorylation of STAT3, thereby enhancing the radiosensitivity of cancer stem cells [32,60,87]. Similarly, Aspirin exerts preventive and therapeutic effects by inhibiting STAT3 expression and phosphorylation [33,88]. Another compound, K-80003, a derivative of Sulforaphane, also inhibits colorectal cancer development through the suppression of STAT3 phosphorylation [89]; Meloxicam is recognized as a potential inhibitor of STAT3 due to its inhibitory effects on STAT3 activation [90]. However, caution is warranted with the long-term use of Indomethacin, which can activate STAT3 and inhibit the production of TNF-α and IFN-γ, potentially worsening the prognosis for hepatocellular carcinoma [91]. This highlights the complexity of NSAIDs’ mechanisms of action, suggesting that while they may offer some anti-tumor benefits, their use as cancer treatments should be approached with care in clinical settings.

#### 3.1.4. Mammalian Target of Rapamycin (mTOR)

As a crucial serine/threonine protein kinase, mTOR plays a significant role in cellular processes such as growth, proliferation, and metabolism [92], making it a target for NSAID action (Figure 2F,G). Early studies have shown that Aspirin is capable of inhibiting mTOR and protein kinase B (Akt) via adenosine monophosphate-activated protein kinase (AMPK)-dependent and non-AMPK-dependent pathways, inducing cellular autophagy, and thereby acting as an anticancer agent [34]; similarly, Meloxicam activated AMPK and inhibited mTOR phosphorylation in hepatocellular carcinoma cells [53]. Akt, another serine/threonine kinase, is closely linked to cancer development. Research by Mei et al. demonstrates that Aspirin-activated AMPK can upregulate mTOR and the anti-apoptotic protein MCL-1, which may diminish its anticancer effects [93]. While both mTOR and Akt inhibit the downstream effector S6K1, Aspirin inhibits Akt, whereas Akt phosphorylation can paradoxically enhance mTOR activation. Additionally, Aspirin inhibits the expression of the downstream sterol-regulatory element binding protein (SREBP) by targeting the Akt/mTOR signaling pathway, thereby promoting ferroptosis in response to RSL [35]. Beyond its effects on the Akt/mTOR axis, Aspirin also inhibits the activation of upstream Phosphatidylinositide 3-kinases (PIK3), influencing the survival of colorectal cancer cells (CRC) [36]. Although the mechanisms by which Aspirin inhibits the mTOR signaling pathway are relatively well understood, the challenges encountered during this inhibition remain largely unexplored.

#### 3.1.5. Neuraminidase-1 (Neu-1)

Neu-1, an enzyme present in lysosomes, is closely related to ligand-induced receptor activation, and abnormal receptor activation may further promote the transduction of downstream signals, thereby promoting cancer development [94]. Recent studies have demonstrated that both Aspirin and Celecoxib inhibit Neu-1 activity, leading to the induction of apoptosis in pancreatic cancer cells (Figure 2H) [55].

### 3.2. Meloxicam and Other NSAIDs Mediate Cell Behavior

**Figure 3:**
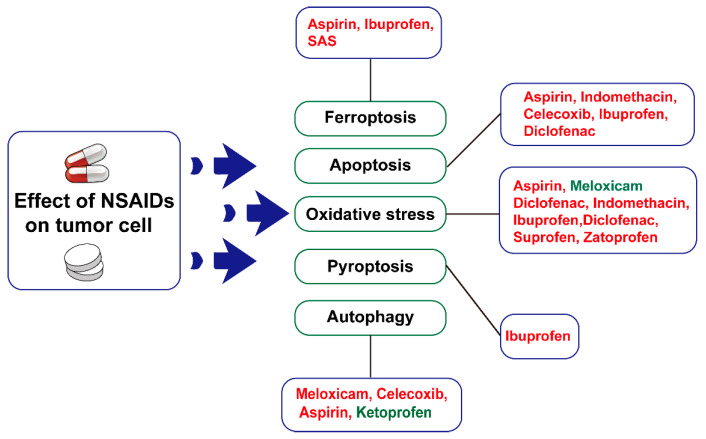
Meloxicam and other NSAIDs mediate cell behavior. Drugs marked in red font represent that the drug is a facilitator of a cellular behavior; while drugs marked in green font express an inhibitory effect on a cellular behavior.

In addition to protein targets, Meloxicam and traditional NSAIDs modulate a variety of cellular behaviors: oxidative stress, apoptosis, cellular focal death, iron death, and autophagy (Figure 3). These are discussed below:

#### 3.2.1. Oxidative Stress

Research indicates that NSAIDs, such as Aspirin and its derivatives [37], Meloxicam [95], Diclofenac [96], and Indomethacin [51], can disrupt the balance of redox reactions, leading to oxidative stress and the production of ROS, which in turn trigger cell death [97]. Furthermore, NSAIDs induce endoplasmic reticulum (ER) stress, which leads to the activation of death receptor 5 (DR5) and the pro-apoptotic protein BID, both of which play significant roles in tumor suppression [98]. NSAIDs also play a supportive role in cancer therapy. For instance, oxidative stress induced by Indomethacin enhances DR5 signaling and promotes apoptosis through the TRAIL-DR5 pathway, thereby improving T-cell therapy outcomes [51]. Similarly, Diclofenac has been shown to amplify the anticancer effects of sorafenib by increasing oxidative stress [98]. However, some studies suggest that NSAIDs may also reduce oxidative stress, offering protective effects, as seen with Meloxicam’s protective role for peripheral blood stem cells [54]. The relationship between oxidative stress and its protective effects remains unclear, indicating a complex interplay. While NSAIDs can induce apoptosis in tumor cells, the mechanisms behind this induction are intricate and partially linked to oxidative stress. Mitochondria serve as both the site of ROS production and the central hub for apoptosis. It has been found that the deletion of SMAC in mitochondria can diminish the tumor-inhibitory effects of NSAIDs by blocking apoptosis [99]. Furthermore, proline dehydrogenase/proline oxidase (PRODH/POX), localized in mitochondria, contributes to oxidative stress by degrading proline to generate ROS. Several NSAIDs, including Ibuprofen, Indomethacin, Diclofenac, Sulforaphane, and Zaltoprofen, have been found to induce apoptosis in tumor cells by upregulating PRODH/POX expression and increasing ROS production [42].

#### 3.2.2. Apoptosis

Apoptosis is a genetically regulated programmed death, which is essential for maintaining the homeostasis of body tissues and eliminating harmful or unnecessary cells from organisms; therefore, apoptosis has become one of the important mechanisms in the fight against cancer. However, cancer cells often evade apoptosis, allowing for uncontrolled proliferation. The mechanisms behind this evasion can be broadly classified into three categories: (1) disruption of the balance between pro-apoptotic and anti-apoptotic proteins, (2) reduced function of cysteine asparaginase, and (3) impaired signaling at death receptors [100]. The B Cell Lymphoma 2 (Bcl2) family of proteins is one of the key players in the development of apoptosis and contains both pro- and anti-apoptotic proteins that balance the decision between cell life and death [101]. During tumor progression, cancer cells often down-regulate pro-apoptotic proteins, such as Bax [102] while up-regulating anti-apoptotic proteins like Bcl-2 and Bcl-XL [103,104], thereby protecting themselves from apoptosis. Additionally, low levels or functional impairments of caspases, which are critical for initiating and executing apoptosis, are commonly observed in various cancers. For instance, caspase-9 is often expressed at low levels in colorectal cancers [105], while reduced expression of caspase-3, -8, and -10 also contributes to cancer development [106,107]. Impaired signaling of death receptors and ligands for death receptors, one of the key players in the extrinsic pathway of apoptosis, can help cancer cells escape apoptosis: dysregulation of DR4 and DR5 can promote cervical carcinogenesis [108]. Consequently, targeting apoptosis has emerged as a promising strategy for cancer treatment. Interestingly, some NSAIDs also influence apoptosis. Meloxicam, Aspirin, Indomethacin, and Celecoxib can down-regulate Bcl-2 expression and up-regulate Bax expression, thereby inducing apoptosis in cancer cells [16,26,109,110]. Additionally, Aspirin, Celecoxib, Ibuprofen, and Diclofenac can activate caspases 3, 7, and 8, promoting apoptosis in breast cancer cells [41,56,61]. Furthermore, Indomethacin has been shown to enhance DR5 signaling, improving T-cell therapy outcomes in B-cell lymphoma models [51].

#### 3.2.3. Pyroptosis

Pyroptosis is a lytic and inflammatory programmed cell death that is typically triggered by the inflammasome and executed by gasdermin proteins, thereby inducing the release of cytokines, such as IL-1β and IL-18 [111]. Unlike apoptosis, which primarily regulates cell death, pyroptosis plays a significant role in the immune response. Under normal physiological conditions, moderate pyroptosis is essential for defending against pathogen infections. However, excessive or persistent pyroptosis can lead to chronic inflammation, which may contribute to disease progression. In the context of tumor development, while spontaneous pyroptosis in cancer cells can exhibit anti-tumor effects [112], the cytokines released during this process can also promote tumor invasion and increase the likelihood of metastasis [113]. In the face of the double-edged sword of pyroptosis, it is undoubtedly necessary to be cautious in treating cancer by targeting pyroptosis. NSAIDs, known for their anti-inflammatory properties, raise the question of whether they can exert anti-tumor effects by inhibiting inflammation and inducing pyroptosis in cancer cells [43].

#### 3.2.4. Ferroptosis

Ferroptosis is a newly discovered form of cell death characterized by lipid peroxidation. Like apoptosis and pyroptosis, ferroptosis contributes to maintaining homeostasis in the body under physiological conditions. This process can inhibit tumor progression by regulating the growth and proliferation of specific cancer cells [114]. Consequently, ferroptosis presents significant potential for cancer treatment. Numerous studies have demonstrated that drugs such as Aspirin [35,115], Ibuprofen [116], and SAS [48,49,50] can induce ferroptosis in cancer cells, thereby playing a role in both prevention and treatment.

#### 3.2.5. Autophagy

Autophagy is a cellular behavior that can promote the degradation and recycling of cellular substances, serving as a crucial pathway for maintaining body homeostasis. It has protective effects against disease development. In cancer, early studies suggested that autophagy acts as a tumor suppressor [117]. However, more recent research indicates that autophagy can also promote cancer progression by affecting the proliferation, metabolism, and immune microenvironment of tumor cells [118,119,120]. Targeting autophagy in cancer therapy presents significant challenges. Nevertheless, certain NSAIDs have been shown to inhibit tumor growth by modulating autophagic processes. For instance, selective COX-2 inhibitors such as Meloxicam and Celecoxib can induce protective autophagy [16,58]. Additionally, Aspirin has been found to trigger Beclin-1-dependent autophagy in liver cancer cells [121]. Ketoprofen can inhibit breast cancer growth by down-regulating autophagy-related proteins such as LC3-II, Beclin-1, and ATG7 [52]. This highlights the dual role of autophagy in cancer development.

### 3.3. Meloxicam and Other NSAIDs Mediate Signaling Pathways

**Figure 4:**
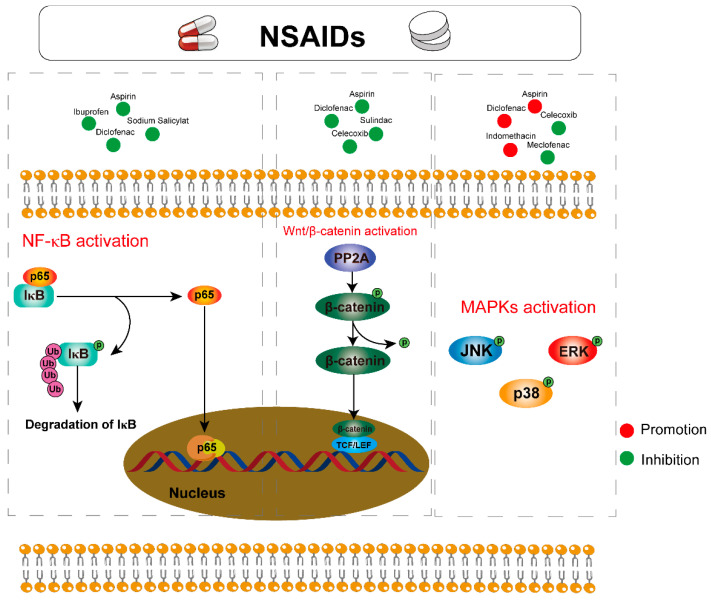
Effects of Meloxicam and other NSAIDs on activation and transduction of NF-κB, MAPKs, and Wnt/β-Catenin signaling pathways.

In addition to protein targets and cell behaviors, Meloxicam and traditional NSAIDs can modulate many different signaling pathways, such as NF-κB, MAPK and Wnt/β-Catenin (Figure 4). These are discussed below.

#### 3.3.1. Nuclear Factor Kappa-B (NF-κB) Pathway

As a transcription factor that is often activated during inflammatory and immune responses, The nuclear factor kappa-B (NF-κB) is activated in many cancers. It has been pointed out that NF-κB is closely related to tumor development. The inflammatory microenvironment surrounding tumor cells increases the accumulation of ROS and RONS, which elevate the mutation rate and promote tumor development [3]. Additionally, immune cells such as macrophages and neutrophils can enhance cancer cell proliferation by activating NF-κB, leading to the expression of pro-inflammatory cytokines like TNF-α, IL-1β, and IL-6 [122,123]. Furthermore, NF-κB promotes angiogenesis by regulating vascular growth factors, facilitating tumor invasion [2,124].

Given the crucial role of NF-κB in tumorigenesis and progression, it presents a potential target for cancer treatment and prevention. Research indicates that Aspirin and other NSAIDs, including H2S-NSAIDs, Diclofenac, Ibuprofen, and Sodium Salicylate, can inhibit NF-κB activation by preventing the degradation of IκBα and the activation of P65 [20,63,115,125].

#### 3.3.2. Mitogen-Activated Protein Kinases (MAPKs) Pathway

MAPKs constitute a family of serine/threonine kinases, can respond to diverse stimuli and facilitate the transmission of signals from the cell membrane to the nucleus, thereby regulating critical biological processes such as proliferation, differentiation, apoptosis, and immune responses [126]. Furthermore, they are often overactivated in pathological conditions including inflammation and tumorigenesis. Studies have shown that the activation of JNK, p38 and ERK MAPK signal transduction can promote a series of processes such as tumor cell growth, cancer cell invasion, metastasis and angiogenesis, thus contributing to tumor progression, and the use of inhibitors partially reverses their pro-tumor effects [127]. The current study suggests that NSAIDs can inhibit tumor development by targeting the MAPKs signaling pathway.

For instance, Aspirin [38] and Indomethacin [128] have been noted to promote tumor cell apoptosis through the activation of p38 and JNK. The use of SB203580, a p38 inhibitor, significantly reduced the tumoricidal effects of Indomethacin [129]. Celecoxib also inhibits ERK phosphorylation while enhancing p38 and JNK signaling, leading to reduced cell growth and increased apoptosis [57,130,131]. In addition, Meclofenac and Diclofenac have been shown to enhance the sensitivity of cancer cells by inhibiting the phosphorylation of p38 and ERK [67,131].

#### 3.3.3. Wnt/β-Catenin Pathway

As a highly conserved signaling pathway, the Wnt/β-catenin pathway regulates various processes of cell proliferation, differentiation, and apoptosis, thereby playing a crucial role in the occurrence and progression of many cancers [132]. For example, Wnt5a is able to secrete the chemokine CCL2 through the Wnt5a-CaMKII-ERK pathway, altering the tumor microenvironment and promoting tumor cell proliferation and migration [133]. Wnt2 can inhibit the anti-tumor effects mediated by dendritic cells through the SOCS3/p-JAK2/p-STAT3 pathway [134]. Additionally, the deletion of the oncogene P53 leads to aberrant Wnt signaling activation, further promoting the metastatic spread of breast cancer associated with systemic inflammation [135]. This pathway is also implicated in the development of lung and gastric cancer [136], as well as drug-resistance in pancreatic cancer [137] and the development of drug-resistant pancreatic cancer, which are all important factors in the development of breast cancer [138].

Studies have shown that NSAIDs, including Aspirin [39], Sulindac [45], Diclofenac [59], and Celecoxib [59], can target the Wnt/β-catenin signaling pathway. Diclofenac and Celecoxib induce the phosphorylation of β-catenin and inhibit the Wnt/β-catenin/TCF signaling pathway, ultimately reducing tumor cell growth and migration [59]. Aspirin can also inactivate protein phosphatase 2A (PP2A) and promote the degradation of β-catenin, thereby suppressing the Wnt signaling pathway [40]. In addition, Sulindac is also able to inhibit the Wnt/β-catenin/TCF signaling pathway by activating cGMP/PKG, which in turn inhibits tumor growth [46,139].

## 4. Aspirin Prevents and Treats Cancer and CVD

NSAIDs are commonly used as the first line of treatment for pain and have been widely employed in the prevention and treatment of various diseases globally. Given that CVD is one of the leading causes of death worldwide, the relationship between NSAIDs and CVD has garnered significant attention. Among the many NSAIDs, Aspirin has been extensively studied for its effects on CVD [139,140].

### 4.1. Lipoxin A4 (LXA4)

LXA4 is a metabolite of ARA, which is mainly produced by immune cells and inhibits inflammation by various signaling pathways, including MAPKs, NF-κB, and PIK3K/Akt, thus exerting anti-inflammatory and immunomodulatory effects [141,142]. Due to these properties, LXA4 demonstrates significant preventive and protective effects against CVD-related conditions such as myocardial injury [143], infarction [144], atherosclerosis [145], and cerebrovascular injury [146]. Research indicates that a deficiency in LXA4 can worsen cardiac and renal dysfunction, leading to myocardial damage [147]. Aspirin amplifies its anti-inflammatory effect by inducing COX acetylation to generate LXA4; moreover, Aspirin-induced LXA4 can target the Fpr2 receptor [144], helping to alleviate atherosclerosis and systemic inflammation.

### 4.2. Phosphatidylinositol-3-kinase/Protein Kinase B (PI3K/Akt) Signaling Pathway

As mentioned above, mTOR is frequently abnormally activated in tumors, and the PI3K/Akt signaling pathway, which is upstream of mTOR, is also implicated in tumor development. Phosphatidylinositol kinase (PI3K), when activated, is able to activate Akt by phosphorylating it; activated Akt is able to participate in cell survival, growth, differentiation, and other processes by regulating downstream targets. This regulatory mechanism often becomes dysregulated in tumor pathology [148]. Targeting the PI3K/Akt signaling pathway has emerged as a promising strategy for anti-cancer therapy. In addition, the PI3K/Akt signaling pathway is often abnormally activated in the pathology of CVD [149]. Inhibiting its activation can ameliorate conditions such as inflammation [150], heart disease [151], and atherosclerosis [152]. It has been found that Aspirin can down-regulate the level of Akt phosphorylation, thereby reducing cardiac interstitial fibrosis [153] and inhibiting platelet aggregation [154], which contributes to achieving anti-atherosclerotic effects [155].

### 4.3. The Related Acetyltransferases and Deacetylases

The addition of acetylated residues to lysine drives protein acetylation, and various acetyltransferases and deacetylases have been shown to be involved in tumor development. In renal carcinoma, low expression of the histone acetyltransferase MOF has been characterized as a potential tumor suppressor. Evidence demonstrates that overexpression of MOF can increase the expression of SIRT1 at both mRNA and protein levels, subsequently inhibiting cancer cell proliferation and migration [156]. Conversely, in hepatocellular carcinoma, increased levels of the histone acetyltransferase hMOF are associated with enhanced cancer cell invasion and metastasis [157]. In addition, acetyltransferases such as NAT10 and KAT6A promote tumor development by regulating the Wnt/β-catenin and PI3K/Akt signaling pathways [158,159]. This suggests that acetyltransferases may serve as new anti-tumor targets, and the use of acetyltransferase inhibitors indicates that targeting these enzymes can exhibit partial anti-tumor effects. Aspirin has been reported to inhibit the acetyltransferase activity of P300 [160] and SIRT1 [62], contributing to its anti-tumor activity. Ghosh, Asish K noted that acetyltransferase P300 plays a significant role in the development and incidence of CVD, and its inhibitors can target P300 to improve related pathological processes [161]. Furthermore, Peng Li et al. highlighted that lysine acetyltransferases and deacetylases could be potential targets for CVD treatment [162]. Aspirin exerts protective effects on the cardiovascular system by inhibiting the activity of acetyltransferase P300 [160]. Additionally, AEE, a derivative of Aspirin, has been shown to inhibit platelet aggregation by modulating SIRT1 [154].

### 4.4. The NOD-like Receptor Thermal Protein Domain Associated Protein 3 (NLRP3)

NLRP3 is an inflammatory complex that can be activated by various stimuli. Upon activation, NLRP3 interacts with the apoptosis-associated speck-like protein containing a CARD (ASC) to form a large oligomeric structure, which serves as a platform for recruiting and activating caspase-1. This activation leads to the cleavage of Gasdermin D, driving pyroptosis and the secretion of caspase-1 and IL-1β [163]. Abnormal secretion of caspase-1 and IL-1β can trigger inflammatory responses [164]. Inhibiting NLRP3 can alleviate many pathological processes associated with immune dysregulation. Studies have shown that Aspirin [165], Indomethacin [166] and some novel NSAIDs [167,168] can inhibit the expression of proteins related to the NLRP3 signaling pathway, demonstrating anti-inflammatory effects. In the body, NLRP3 is typically maintained in a dynamic balance; disruption of this balance can lead to excessive NLRP3 activation, promoting the occurrence and progression of CVD [163]. The inhibitory effect of Aspirin on NLRP3 is also evident in CVD, as it helps prevent the disease by ameliorating endothelial cell damage [169].

## 5. Conclusions and Future Perspectives

The complex relationship between inflammation and disease pathogenesis highlights the pivotal function of immune responses in the development of both cancer and CVD. While acute inflammation serves a protective function, chronic and dysregulated inflammatory responses can result in adverse outcomes, including tumor development and CVD. This review highlights the potential of NSAIDs, particularly Meloxicam, as therapeutic agents that target inflammation—a shared risk factor for both cancer and CVD. In recent years, numerous studies have investigated the role of Meloxicam in cancer treatment; however, research regarding its impact on CVDs remains limited. As one of the NSAIDs, like Aspirin, Meloxicam can also target the PI3K/Akt signaling pathway, SIRT1, and mTOR. Therefore, it is plausible to hypothesize that Meloxicam may serve as a potential therapeutic agent for the prevention and management of CVDs. Nonetheless, this hypothesis necessitates further validation in future studies.

NSAIDs operate through both COX-2-dependent and COX-2-independent pathways, thereby offering a multifaceted approach to the mitigation of inflammation. However, conventional NSAIDs targeting COX-2 often result in the inadvertent modulation of COX-1, which plays a protective role in various physiological processes. This dual inhibition may result in adverse effects, which gives rise to concerns regarding the safety profiles of these medications. Although selective COX-2 inhibitors have been developed to minimize COX-1-related side effects, they still pose safety challenges that necessitate careful consideration in clinical use.

As our comprehension of the intricate interrelationship between inflammation, cancer, and cardiovascular health advances, there is a growing recognition of the need to explore COX-independent mechanisms of action for NSAIDs. This exploration is vital for the development of innovative therapeutic strategies that effectively target inflammation without compromising patient safety. By identifying and validating alternative molecular targets beyond COX-2, researchers can pave the way for the design of novel anti-tumor agents and therapeutics for CVD that harness the anti-inflammatory properties of NSAIDs while minimizing associated risks.

The prospect of NSAIDs as integral components of treatment regimens for cancer and CVD is promising, particularly as they may offer synergistic effects when combined with existing therapies. Future research should focus on elucidating the specific COX-independent pathways activated by NSAIDs and their implications for clinical outcomes. Additionally, the development of biomarkers to predict patient responses to NSAID therapy could enhance personalized treatment approaches, optimizing efficacy while reducing the likelihood of adverse effects.

In summary, the ongoing investigation into the COX-independent pathways of NSAIDs represents a significant advancement in our understanding of their therapeutic potential. By addressing the challenges associated with inflammation as a common risk factor for cancer and CVD, this research lays the groundwork for innovative drug development that balances efficacy and safety. Ultimately, the insights gained from exploring these pathways may lead to transformative strategies in the prevention and treatment of these interconnected diseases, improving patient outcomes and quality of life.

## Data Availability

Data are contained within the article.
